# Primary small cell carcinoma of the esophagus: clinicopathological and immunohistochemical features of 21 cases

**DOI:** 10.1186/1471-2407-7-38

**Published:** 2007-03-03

**Authors:** Jing-Ping Yun, Mei-Fang Zhang, Jin-Hui Hou, Qiu-Hong Tian, Jia Fu, Xiao-Man Liang, Qiu-Liang Wu, Tie-Hua Rong

**Affiliations:** 1 State Key Laboratory of Oncology in Southern China, Cancer Center, Sun Yat-Sen University, Guangzhou, China; 2 Department of Pathology, Cancer Center, Sun Yat-Sen University, Guangzhou, China; 3 Thoracic Surgery, Cancer Center, Sun Yat-Sen University, Guangzhou, China

## Abstract

**Background:**

Primary small cell carcinoma (SCC) of the esophagus is a rare and aggressive tumor with poor prognosis. In this study, we report the clinicopathological characteristics of 21 cases of small cell carcinoma of the esophagus treated at the Cancer Center of Sun Yat-Sen University, with particular focus on the histologic and immunohistochemical findings.

**Methods:**

Twenty-one patient records were reviewed including presenting symptoms, demographics, disease stage, treatment, and follow-up. Histologic features were observed and immunohistochemical detection of cytokeratin (CK), epithelial membrane antigen (EMA), neuron specific enolase (NSE), synaptophysin (Syn), chromogranin A (CgA), neuronal cell adhesion molecules (CD56), thyroid transcriptional factor-1 (TTF-1) and S100 protein (S100) was performed.

**Results:**

The median age of patients in the study was 56 years, with a male-to-female ratio of 3.2:1. Histologically, there were 19 "homogenous" SCC esophageal samples and 2 samples comprised of SCC and well-differentiated squamous cell carcinoma. The percentages of SCC samples with positive immunoreactivity were Syn 95.2%, CD56 76.2%, TTF-1 71.4%, NSE 61.9%, CgA 61.9%, CK 57.1%, EMA 61.9%, and S100 19.0%, respectively. The median patient survival time was 18.3 months after diagnosis. The 2-year survival rate was 28.6%.

**Conclusion:**

Our study suggests that esophageal SCC has similar histology to SCC that arises in the lung compartment, and Chinese patients have a poor prognosis. Higher proportion of positive labeling of Syn, CD56, CgA, NSE, and TTF-1 in esophageal SCC implicate that they are valuably applied in differential diagnosis of the malignancy.

## Background

Primary small cell carcinoma (SCC) of the esophagus is a relatively rare malignancy, accounting for 0.05 – 4% of all esophageal malignancies [1]. It is a highly aggressive tumor associated with a poor prognosis, similar to SCC that arises in the lung [2] and other extrapulmonary organs, including breast [3], ovary [4], uterine cervix [5], liver [6], salivary gland [7], stomach [8], colon [9], prostate [10], urinary bladder [11], and kidney [12]. Histologically, SCC is characterized by neuroendocrine-like architectural patterns, including nested and trabecular growth with common features including peripheral palisading and rosette formation in the tumors [2-12]. Some SCC cases include carcinomas such as squamous cell carcinoma and adenocarcinoma [2]. Analogous to small cell lung cancer, diagnosis of esophageal SCC is aided by immunohistochemical staining for common neuroendocrine markers, including Syn, CgA and NSE. Recently, CD56 (neuronal cell adhesion molecule) and TTF-1 (Thyroid Transcriptional Factor-1) were also reported to be high positivity in small cell carcinoma arising in different organs and thought to be useful markers for diagnosis of the tumor.

A total of 4050 patients with esophageal malignancies were seen at the Cancer Center of Sun Yat-Sen University during the period between 1989 to 2005, and 21 of these cases were diagnosed as esophageal SCC. The conclusions of this study focus on the histologic and immunohistochemical findings of these 21 cases.

## Methods

Between 1989 and 2005, a total of 4050 Chinese patients with esophageal carcinoma were seen in the Department of Thoracic Surgery Unit of the Cancer Center of Sun Yat-Sen University. Of these patients, a total of 21 cases were diagnosed as small cell carcinoma of the esophagus, using histological criteria for esophageal small cell carcinoma [13] and pulmonary small cell carcinoma [14] prepared by the World Health Organization. The clinical, pathologic, and radiographic records were reviewed. The reported data includes gender, age, initial symptoms and duration, pathological examination, lymph node metastases, upper gastrointestinal barium studies, endoscopic biopsy, Doppler ultrasound of the abdomen, chest radiography or computed tomography scan of the thorax, treatment, and follow-up. All of the human specimens in the study were approved by the Independent Ethics Committee of the Cancer Center of Sun Yat-Sen University.

For immunohistochemistry studies, a labeled-streptavidin-biotin (LAB-SA) method was performed with Histostain^®^-*P*lus Bulk Kit Zymed^® ^2^nd ^generation LAB-SA detection system (CAT. NO. 85-9043, Zymed Laboratories, CA). Immunological markers included pancytokeratin (CK; mouse monoclonal antibody; Clone:AE1/AE3; Cat No. 18-0132; Zymed, CA), epithelial membrane antigen (EMA; mouse monoclonal antibody; Clone:MC-5; Cat No. MU182-UC; BioGenex, CA), neuron specific enolase (NSE; mouse monoclonal antibody; Clone:NSE-1G4; Cat No. 18-01963; Zymed, CA), synaptophysin (Syn; mouse monoclonal antibody; Clone:snp88; Cat No. MU363-UC; BioGenex, CA), chromogranin A (CgA; mouse monoclonal antibody; Clone:LK2H10; Cat No. MU126-UC; BioGenex, CA), neuronal cell adhesion molecule (CD56; mouse monoclonal antibody; Clone:123C3; Cat No.18-0152; Zymed, CA), Thyroid Transcriptional Factor-1 (TTF-1; mouse monoclonal antibody; Clone:8G7G3/1; Cat No.18-0221; Zymed, CA) and S100 protein (S100; mouse monoclonal antibody; Clone:4C4.9; Cat No. Z2055; Zeta, CA). All the primary antibodies were ready to use without dilution. Each paraffin-embedded tissue section (4 μm in thickness) was deparaffinized, hydrated, and incubated in 3% H_2_O_2 _and microwaved for 3 minutes to block endogenous peroxidase activity. The tissue sections were subjected to antigen retrieval by microwaving in 10 mM citrate buffer for 30 min. The sections were incubated with serum blocking solution (Reagent A) for 10 minutes to block nonspecific binding and then with the primary antibodies in moist chamber for 60 minutes. After rinsed with PBS for 2 minutes, the sections were incubated with the biotinylated secondary antibody (Reagent B) for 10 minutes and rinsed with PBS. The sections followed by incubation with enzyme conjugate (Reagent C) for 10 minutes. Subsequently, the sections were stained with DAB and counterstained with hematoxylin. Serum blocking solution (Reagent A) in place of the primary antibody was used as a negative control. CK, EMA, NSE, Syn, and CgA showed positive immunoreactivity in the cytoplasms of the cells. TTF-1 showed positive immunoreactivity in the nuclei of the cells. CD56 showed positive immunoreactivity in the membranes of the cells.S-100 showed positive immunoreactivity in the nuclei and cytoplasms of the cells. Immunostaining labeling intensities were defined as: +, less than 10% of the positive tumor cells; ++, 10%–50% of the positive tumor cells; +++ more than 50% of the positive tumor cells; - negative labeling.

Statistical analysis was performed on relationship between clinical parameters including gender, age, tumor location, tumor size, and stage, 8 immunohistochemical markers and patient prognosis by Two-Sample Kolmogorov-Smirnov Test using software of SPSS 10.0 (SPSS Inc., Chicago, IL). Eighteen died cases of 21 patients with SCC were used to compare with their clinicopathological parameters. In these parameters, two groups each one was divided for comparison, including gender: male and female; age: ≤50 and >50 years old; tumor size: < 5 cm and ≥5 cm in diameter; tumor location: locations in upper one-third or middle one-third and location in lower one-third; stage: stages T1 or T2 and stages T3 or T4; immunohistochemistry data: positive labeling marked as + or ++ or +++ and negative labeling marked as - for each marker. The statistically significant difference was set P < 0.05 (two-sided probability).

## Results

### Clinical features

Between 1989 and 2005, twenty-one Chinese patients were diagnosed with primary SCC, representing 0.05% (21/4050) of all patients with esophageal malignancies seen in our hospital. Clinicopathological data were summarized in Table 1. The median age of the patients at the time of diagnosis was 56 years, ranging from 30 to 76 years. Sixteen patients were men, with a male-to-female ratio of 3.2:1. The most common initial symptoms were dysphagia (18 cases, 85.7%) and retrosternal pain (3 cases, 14.3%). The duration of symptoms varied from 10 days to 9 months with a mean time of 2 months. The tumors were located in the upper third of esophagus in 1 case, the middle third in 12 cases, and the lower third in 8 cases. Upper gastrointestinal barium examination showed medullary morphology of the tumor in 18 out of 21 cases, ulceration in 2 cases, and a mushroom-like appearance in 1 case. The mean tumor size was 5.8 cm long, ranging from 1.2 cm to 15.5 cm.

### Pathologic characteristics

Endoscopic biopsy results were available for 19 SCC patients. Upon diagnosis, 5 patients presented with liver metastasis, and 1 patient presented with thyroid gland metastasis. Twelve patients underwent surgery, and lymph node metastases were found in 8 cases, while 2 cases showed SCC mixed with squamous cell carcinoma.

**Figure 1:**
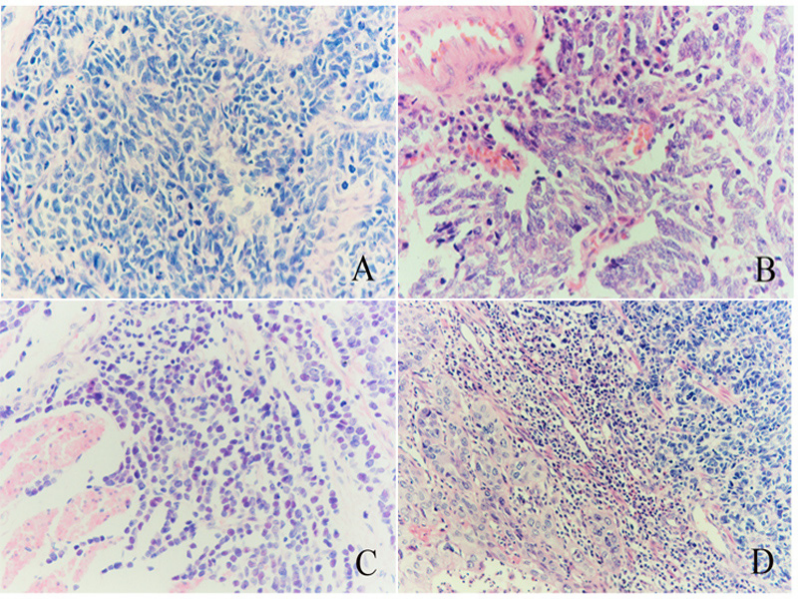
Hematoxylin and eosin-stained sections showing morphology of esophageal SCC morphology. Nested or organoid growth pattern was the most common (A). Sheet-like growth was a dominant pattern in 3 cases (B). In one case, we observed marked cellularity similar to lymphoma (C). Combined SCC of the esophagus with well-differentiated squamous cell carcinoma (D). (Mag. × 400).

A total of 2 – 8 hematoxylin and eosin-stained sections were examined for each patient. All tumors conformed to 2000 WHO histological criteria for small cell carcinoma of the esophagus. Histological sections were referenced using 2004 WHO histological criteria for pulmonary small cell carcinoma, consisting of small, round, ovoid or spindle-shaped cells with scant cytoplasm, ill-defined cell borders, finely granular nuclear chromatin, and absent or inconspicuous nucleoli (Figure 1). A nested or organoid growth pattern was commonly seen, and peripheral palisading of tumor cell nests was observed in most cases (Figure 1A). Sheet-like growth was a dominant growth pattern in 3 cases (Fig. 1B). In one case, morphology indicated that lymphoma was present (Figure 1C). A total of 19 "homogenous" SCC cases and 2 cases with SCC mixed with well-differentiated squamous cell carcinoma were found (Figure 1D). In the 2 cases of SCC and squamous cell carcinoma, no transitional lesion was found between the two components.

### Immunohistochemistry

**Figure 2:**
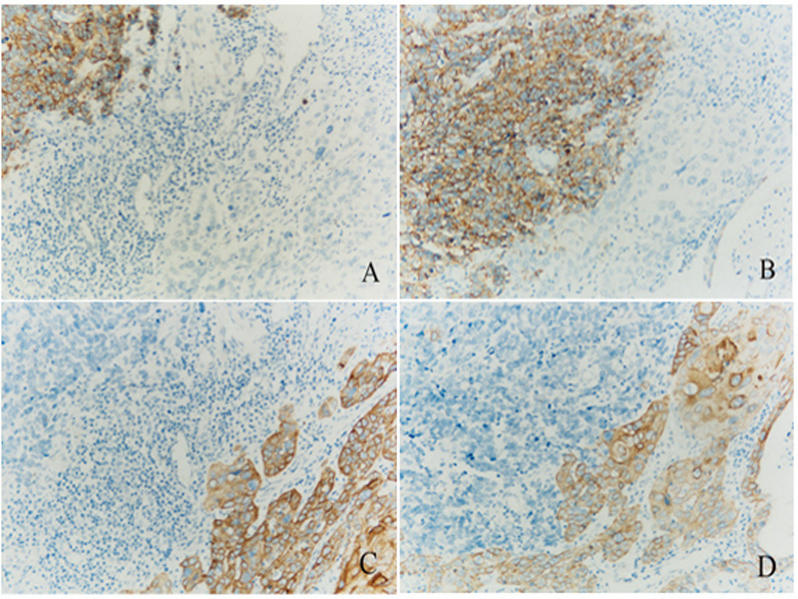
Immunohistochemical staining for CD56, Syn, CK, and EMA in the combined SCC case. CD56 (A) and Syn (B) immunopositivity was observed in the SCC area, and CK (C) and EMA (D) immunopositivity was observed in the regions of squamous cell carcinoma (Mag. × 400).

Immunohistochemical study information was available for all 21 cases and was shown in Table 2 and Figure 2. Staining for eight immunological markers (CK, EMA, NSE, Syn, CgA, CD56, TTF-1 and S-100) was performed on all of the cases. Immunological reactivity of the samples was Syn 95.2%, CD56 76.2%, TTF-1 71.4%, NSE 61.9%, CgA 61.9%, CK 57.1%, EMA 61.9%, and S100 19.0%. In the two cases of combined squamous cell carcinoma, the squamous cell carcinoma compartment showed positive CK and EMA staining, but negative neuroendocrine marker staining. The small cell carcinoma compartment showed positive neuroendocrine staining and negative epithelial labeling (Figure 2).

### Treatment

Nineteen patients underwent various treatments, including resection, chemotherapy, radiotherapy and combined therapy, summarized in Table 1. Of the 19 patients that obtained treatment, 7 patients underwent treatment with chemotherapy and surgery, 3 patients underwent chemotherapy and radiotherapy, 2 patients underwent resection, chemotherapy and radiotherapy, 2 patients underwent resection and radiotherapy, 2 patients underwent resection alone, 2 patients underwent chemotherapy alone, and 1 patient underwent radiotherapy alone.

### Follow-up

The median survival of the 21 patients was 18.3 months after diagnosis. Follow-up information was summarized in Table 1. Of the 19 patients that received treatment, the median survival was 19.6 months, and the median survival of the 2 patients without anti-tumor treatment was 6 months. The longest survival time was 71 months, and the shortest survival time was 3 months. The 2-year survival rate was 28.6% (6/21) and the 5-year survival rate was 4.8% (1/21). Six patients died in 6 months (6/21, 28.6%), 9 patients died in one year (9/20, 45%) and 14 patients died in two years (14/19, 73.7%).

### Statistical analysis

The associations of survival time with gender, age, tumor location, tumor size, TNM stage, and the 8 immunohistochemical markers (CK, EMA, NSE, Syn, CgA, CD56, TTF-1 and S-100) were analyzed with nonparametric test (Komolgorov-Smirnov method), and none of them was statistically significant (P < 0.05, two-sided probability)' [see Additional file 1]'.

## Discussion

According to reports from different hospitals, esophageal SCC was a rare disease [15-20]. The present proportion of esophageal SCC at our Cancer Center was about 0.5% of 4010 cases of esophageal malignancies. The incidence of esophageal SCC was much lower than the incidence of invasive lung cancer SCC (15% to 25%) [21]. The clinical features of the patients in this study were consistent with other reports, and dysphagia was the most common presenting symptom in 86% of patients, lasted with 1~ 3 months prior to presentation [15]. The ratio of male-to-female patients was 3.2:1, which is higher than the ratio of 1.3:1 in previous reports [22]. The median age of the patients in our study was 54 years old (ranging from 38 to 76 years old), which was lower than the median age of 63.8 years in one previous report [22], and close to the median age of 58 years in another report [16].

Surgical pathological diagnosis for esophageal SCC was not difficult. Microscopically, esophageal SCC was similar to small cell lung cancer, consisting of round to spindle-shaped cells with scanty cytoplasm, granular nuclei and inconspicuous nucleoli [23-25]. Two cases of esophageal SCC presented with squamous cell carcinoma. Several previous articles reported combined esophageal SCC and squamous cell carcinoma, and it was widely-believed that the histological heterogeneity indicates origin from multipotent reserve cells [26-29]. We did not find any clinical significance related to the histological heterogeneity of the tumors.

Positive labeling with four neuroendocrine markers including Syn, CD56, NSE and CgA was apparent in these cases, which was similar to previous reports of esophageal SCC [28,30] and pulmonary SCC [2]. None of the normal squamous epithelium specimens or heterogeneous components of squamous cell carcinoma showed positive labeling. Thus, Syn, CD56, NSE and CgA were useful markers for differential diagnosis of SCC arising in the lung and the other organs. TTF-1 was a newly described nuclear transcriptional factor expressed in epithelial cells of thyroid and lung and in a high proportion of SCC. In the present study, 71.4% of esophageal SCC cases were positive for TTF-1, which was higher than the previously reported rate [31]. We confirmed that TTF-1 showed higher positive labeling in primary esophageal SCC, suggesting that it was a suitable marker for diagnosis and differentiation of esophageal SCC.

Treatment of esophageal SCC in the present study included surgical resection, chemotherapy, radiotherapy, and combinations of these treatments. We could derive no clear results as to the significance of adjuvant therapies. However, the patients with the longest-term survival (48 and 71 months, respectively) received combination therapies including surgical resection, chemotherapy, and radiotherapy. Due to the small number of cases in our study, statistic analysis was not done. However, follow-up results showed that patients receiving treatment had longer survival (median survival, 19.6 months) than those without treatment (median survival, 6 months). Previous studies reported that surgical resection with systemic chemotherapy, radiotherapy, or combination therapy could produce long-term remission and potential long-term survival in esophageal SCC patients [16,20].

Previous studies from different hospitals showed very poor prognosis for esophageal SCC patients. Investigators at Memorial Hospital [20], Massachusetts General Hospital [17] and M.D. Anderson Cancer Center [16] reported the median survival of esophageal SCC patients to be 7.5 months (range 1–21 months), 7 months (range, 3–17 months) and 12.5 months (range, 5–57 months), respectively. In our study, the median survival was 18 months, ranging from 3 to 71 months, and 28.6% of patients survived longer than 2 years.

The histogenesis of esophageal SCC may be APUD cells or multipotent reserve cells [15,26]. In the present study, observation of dual or multiple cell types, such as the coexistence of squamous elements provides evidence of derivation from multipotent reserve cells. Positive cytokeratin and neuroendocrine labeling supports the theory that SCC arises from the lung, esophagus, and other organs that share histogenesis. Establishing a histogenetic relationship among different organs requires further study, but the current histological and immunological results provide evidence of a common derivation.

## Conclusion

In summary, Chinese esophageal SCC in the present study is relatively rare and has similar histology to SCC that arises in the lung compartment, and patients have a poor prognosis. Higher percentages of positive labeling of Syn, CD56, CgA, NSE, and TTF-1 in esophageal SCC cases implicated that they are valuably applied in differential diagnosis of the malignancy.

## Abbreviations

APUD: amine precursor uptake and decarboxylation; CD56: neuronal cell adhesion molecules; CgA: chromogranin A; CK: cytokeratin; EMA: epithelial membrane antigen; LAB-SA: labeled-streptavidin-biotin; NSE: neuron specific enolase; PBS: phosphate buffer solution; SCC: small cell carcinoma; SqCC: squamous cell carcinoma; Syn: synaptophysin; TTF-1: thyroid transcriptional factor-1.

## Competing interests

The author(s) declare that they have no competing interests.

## Authors' contributions

JPY carried out and coordinated the study, immunohistochemical examinations of tumor specimens and data analysis, and drafted up the manuscript. XML and QLW participated in the study design, immunohistochemical examinations of tumor specimens, and revision of manuscript. THR participated in the study design, interpretation of data, and revision of manuscript. MFZ and QHT participated in interpretation of data, conducted immunohistochemistry and revision of manuscript. JHH and JF participated in conduction of immunohistochemistry. All authors read and approved the final manuscript.

## Pre-publication history

The pre-publication history for this paper can be accessed here:



## Supplementary Material

Additional file 1 Statistics for relationship between clinical parameters, IHC markers and patient prognosis. The data provided represent the statistical analysis of relationship between clinical parameters, IHC markers and patient prognosis made by Two-Sample Kolmogorov-Smirnov Test. Click here for file

## Figures and Tables

**Table 1:** Clinicopathological features of the 21 patients with SCC

Case No.	Gender/Age	Symptoms/duration (months)	Mass location	Gross	Size (cm)	TNM stage or metastasis	Therapy	Follow-up (months/status)
1	M/38	Progressive dysphagia/2	Middle 1/3	Medullary	4	PT3N0M0	S+C+R	48/DOD
2	F/62	progressive dysphagia/1	Middle 1/3	Medullary	3	PT2N1M0 IIb	S+C+R	71/DOD
3	M/62	progressive dysphagia/3	Lower 1/3	Medullary	1.5	PT2N2M0	S	5/DOD
4	M/57	dysphagia/10 days	Lower 1/3	Medullary	1.2	PT2N0M0	S+C	46/alive
5	M/51	dysphagia/1	Upper 1/3	mushroom-like	4	PT3N0M0	S+R	3/DOD
6	M/50	retrosternal complaint/9	Middle 1/3	Medullary	4	PT3N1M1 IV	S+C	13/DOD
7	M/47	dysphagia/2	Middle 1/3	Medullary	10	PT2N0M0	S	3/DOD
8	M/43	progressive dysphagia/3	Lower 1/3	Medullary	7	T4N2M1 IV	S+C	29/DOD
9	F/47	dysphagia/3	Middle 1/3	Medullary	4	PT3N1M0 II	S+C	5/DOD
10	M/45	dysphagia/2 months	Middle 1/3	Medullary	13	PT4N1M0 III	S+C	3/DOD
11	M/68	dysphagia/1	Lower 1/3	Medullary	5	PT2N1M0 II	S+C	15/DOD
12	F/52	dysphagia/2	Lower 1/3	Medullary	3	CT4N1M0	NT	7/DOD
13	M/46	progressive dysphagia/2	Lower 1/3	Medullary	15.5	liver metastasis	C	12/DOD
14	F/63	husk/2 and dysphagia/1	Middle 1/3	Medullary	5.5	thyroid metastasis	C	9/alive
15	M/57	Retrosternal complaint/1	Middle 1/3	Medullary	1.7	liver metastasis	C+R	15/DOD
16	F/76	progressive dysphagia/1	Middle 1/3	Medullary	6	NR	NT	5/DOD
17	M/43	dysphagia and retrosternal pain/2	Middle 1/3	Medullary	3.5	PT3N1M0	S+R	32/DOD
18	M/76	dysphagia/1	Middle 1/3	Ulceration	5	NR	R	28/DOD
19	M/73	Retrosternal complaint/3	Lower 1/3	Medullary	8	PT2N0M0 IIa	C+R	15/DOD
20	M/70	Retrosternal complaint/4	Lower 1/3	Ulceration	9	NR	C+R	13/alive
21	M/54	dysphagia/4	Middle 1/3	Medullary	8	PT3N1M0	C+R	8/DOD

NR, no record; NT, no treatment; S, Resection; C, Chemotherapy; R, Radiotherapy; DOD, Death of disease.

**Table 2:** Immunohistochemical results of 21 SCC cases

Case No	Pathology	CK	EMA	NSE	Syn	CgA	CD56	S-100	TTF-1
1	SCC	++	+++	+++	+++	++	+++	-	+++
2	SCC	+++	++	+	++	-	+	-	-
3	SCC	++	+++	++	+++	+	+++	-	++
4	SCC	+++	++	++	++	++	++	++	+++
5	SCC	++	+++	-	+	-	+++		-
6	SCC	++	++	+	+++	++	++	-	+++
7	SCC	+++	++	+	++	+	+++	+	++
8	SCC	+++	++	+++	++	-	+++	-	++
9	SCC	-	-	+++	+++	++	+++	++	+++
	SqCC	+++	++	-	-	-	-	-	-
10	SCC	-	+	+	+++	+++	-	-	++
11	SCC	-	-	-	++	-	++	-	-
12	SCC	-	-	-	++	-	-	-	+
13	SCC	-	-	++	+++	++	+++	-	-
14	SCC	++	-	+	+++	++	++	-	+++
15	SCC	+	++	-	++	++	+++	-	++
16	SCC	-	-	-	++	-	-	-	++
17	SCC	++	+++	++	+++	+	+++	+	-
18	SCC	-	++	-	-	++	-	-	+++
19	SCC	++	++	-	++	-	+++	-	-
20	SCC	-	-	+	+++	-	++	-	++
21	SCC	-	-	-	+++	+++	-	-	++
	SqCC	+++	-	-	-	-	-	-	-

Total positive cases (%)		12(57.1)	13(61.9)	13(61.9)	20(95.2)	13(61.9)	16(76.2)	4(19.0)	15(71.4)

SCC, small cell carcinoma; SqCC, squamous cell carcinoma. +, less than 10% of the tumor cells were positive; ++, 10%–50% of the tumor cells were positive; +++, more than 50% the tumor cells were positive; -, negative labeling.

## References

[B1] Sasajima K, Watanabe M, Ando T, Hao K, Miyashita M, Yamashita K, Onda M, Takubo K (1990). Serum neuron-specific enolase as a marker of small-cell carcinoma of the esophagus. J Clin Gastroenterol.

[B2] Nicholson SA, Beasley MB, Brambilla E, Hasleton PS, Colby TV, Sheppard MN, Falk R, Travis WD (2002). Small cell lung carcinoma (SCLC): a clinicopathologic study of 100 cases with surgical specimens. Am J Surg Pathol.

[B3] Shin SJ, DeLellis RA, Ying L, Rosen PP (2000). Small cell carcinoma of the breast: a clinicopathologic and immunohistochemical study of nine patients. Am J Surg Pathol.

[B4] Chen L, Dinh TA, Haque A (2005). Small cell carcinoma of the ovary with hypercalcemia and ectopic parathyroid hormone production. Arch Pathol Lab Med.

[B5] Masumoto N, Fujii T, Ishikawa M, Saito M, Iwata T, Fukuchi T, Susumu N, Mukai M, Kubushiro K, Tsukazaki K, Nozawa S (2003). P16 overexpression and human papillomavirus infection in small cell carcinoma of the uterine cervix. Hum Pathol.

[B6] Kim YH, Kwon R, Jung GJ, Roh MH, Han SY, Kwon HC, Jeong JS, Shin TB, Oh JY, Lee KN (2004). Extrapulmonary small-cell carcinoma of the liver. J Hepatobiliary Pancreat Surg.

[B7] Nagao T, Gaffey TA, Olsen KD, Serizawa H, Lewis JE (2004). Small cell carcinoma of the major salivary glands: clinicopathologic study with emphasis on cytokeratin 20 immunoreactivity and clinical outcome. Am J Surg Pathol.

[B8] Kusayanagi S, Konishi K, Miyasaka N, Sasaki K, Kurahashi T, Kaneko K, Akita Y, Yoshikawa N, Kusano M, Yamochi T, Kushima M, Mitamura K (2003). Primary small cell carcinoma of the stomach. J Gastroenterol Hepatol.

[B9] Kim HC, Park SI, Park SJ, Shin HC, Oh MH, Kim HH, Bae WK, Kim IY (2005). Small cell carcinoma of the colon: barium study and CT findings. Br J Radiol.

[B10] Hanazawa K, Higashi N, Kawachi Y, Suzuki F, Ishi K, Fujime M (2005). Small cell carcinoma of the prostate with hypercalcemia. Int J Urol.

[B11] Jones TD, Kernek KM, Yang XJ, Lopez-Beltran A, MacLennan GT, Eble JN, Lin H, Pan CX, Tretiakova M, Baldridge LA, Cheng L (2005). Thyroid transcription factor 1 expression in small cell carcinoma of the urinary bladder: an immunohistochemical profile of 44 cases. Hum Pathol.

[B12] Majhail NS, Elson P, Bukowski RM (2003). Therapy and outcome of small cell carcinoma of the kidney: report of two cases and a systematic review of the literature. Cancer.

[B13] Capella C, Solcia E, Sobin LH, Arnold R, Stanley R Hamilton, Lauri A Aaltonen (2000). Endocrine tumours of the oesophagus. World Health Organization Classification of Tumors, Pathology & Genetics, Tumors of the Digestive System.

[B14] Travis W, Petersen I, Nicholson S, Meyerson M, Hirsch FR, Hanash SM, Pugatch B, Jen J, Geisinger K, Takahashi T, Brambilla E, Fernandez EA, Gazdar A, Capron F, William D Travis, Elizabeth Brambilla, H Konrad Müller-Hermelink, Curtis C Harris (2004). Small cell carcinoma. World Health Organization Classification of Tumors, Pathology & Genetics, Tumors of the Lung, Pleura, Thymus and Heart.

[B15] Law SY, Fok M, Lam KY, Loke SL, Ma LT, Wong J (1994). Small cell carcinoma of the esophagus. Cancer.

[B16] Medgyesy CD, Wolff RA, Putnam JB, Ajani JA (2000). Small cell carcinoma of the esophagus: the University of Texas M. D. Anderson Cancer Center experience and literature review. Cancer.

[B17] Huncharek M, Muscat J (1995). Small cell carcinoma of the esophagus. The Massachusetts General Hospital experience, 1978 to 1993. Chest.

[B18] Bennouna J, Bardet E, Deguiral P, Douillard JY (2000). Small cell carcinoma of the esophagus: analysis of 10 cases and review of the published data. Am J Clin Oncol.

[B19] Isolauri J, Mattila J, Kallioniemi OP (1991). Primary undifferentiated small cell carcinoma of the esophagus: clinicopathological and flow cytometric evaluation of eight cases. J Surg Oncol.

[B20] Nichols GL, Kelsen DP (1989). Small cell carcinoma of the esophagus. The Memorial Hospital experience 1970 to 1987. Cancer.

[B21] Howe HL, Wingo PA, Thun MJ, Ries LA, Rosenberg HM, Feigal EG, Edwards BK (2001). Annual report to the nation on the status of cancer (1973 through 1998), featuring cancers with recent increasing trends. J Natl Cancer Inst.

[B22] Casas F, Ferrer F, Farrus B, Casals J, Biete A (1997). Primary small cell carcinoma of the esophagus: a review of the literature with emphasis on therapy and prognosis. Cancer.

[B23] Mori M, Matsukuma A, Adachi Y, Miyagahara T, Matsuda H, Kuwano H, Sugimachi K, Enjoji M (1989). Small cell carcinoma of the esophagus. Cancer.

[B24] Beyer KL, Marshall JB, Diaz-Arias AA, Loy TS (1991). Primary small-cell carcinoma of the esophagus. Report of 11 cases and review of the literature. J Clin Gastroentero.

[B25] Chen YH (1991). Primary esophageal small cell carcinoma--a report of 10 cases and review of literature. Zhonghua Zhong Liu Za Zhi.

[B26] Ho KJ, Herrera GA, Jones JM, Alexander CB (1984). Small cell carcinoma of the esophagus: evidence for a unified histogenesis. Hum Pathol.

[B27] Osugi H, Takemura M, Morimura K, Kaneko M, Higashino M, Takada N, Lee S, Kinoshita H (2002). Clinicopathologic and immunohistochemical features of surgically resected small cell carcinoma of the esophagus. Oncol Rep.

[B28] Takubo K, Nakamura K, Sawabe M, Arai T, Esaki Y, Miyashita M, Mafune K, Tanaka Y, Sasajima K (1999). Primary undifferentiated small cell carcinoma of the esophagus. Hum Pathol.

[B29] Fujiwara Y, Nakagawa K, Tanaka T, Utsunomiya J, Nishigami T, Uematsu K (1996). Small cell carcinoma of the esophagus combined with superficial esophageal cancer. Hepatogastroenterology.

[B30] Noguchi T, Takeno S, Kato T, Wada S, Noguchi T, Uchida Y, Kashima K, Yokoyama S (2003). Small cell carcinoma of the esophagus; clinicopathological and immunohistochemical analysis of six cases. Dis Esophagus.

[B31] Yamamoto J, Ohshima K, Ikeda S, Iwashita A, Kikuchi M (2003). Primary esophageal small cell carcinoma with concomitant invasive squamous cell carcinoma or carcinoma in situ. Hum Pathol.

