# Supplementary material for: Primary small cell carcinoma of the esophagus: clinicopathological and immunohistochemical features of 21 cases
Source: BMC Cancer. 2007 Mar 3;7:38. doi: 10.1186/1471-2407-7-38 (PMC1829164; doi:10.1186/1471-2407-7-38)
Supplement: Additional file 1 — Statistics for relationship between clinical parameters, IHC markers and patient prognosis. The data provided represent the statistical analysis of relationship between clinical parameters, IHC markers and patient prognosis made by Two-Sample Kolmogorov-Smirnov Test. [file 1471-2407-7-38-S1.doc]

# Primary small cell carcinoma of the esophagus: clinicopathological and immunohistochemical features of 21 cases

Statistics for relationship between clinical parameters, IHC markers and patient prognosis.

**NPar Tests**

**Two-Sample Kolmogorov-Smirnov Test**

**NPar Tests**

**Two-Sample Kolmogorov-Smirnov Test**

**NPar Tests**

**Two-Sample Kolmogorov-Smirnov Test**

**NPar Tests**

**Two-Sample Kolmogorov-Smirnov Test**

**NPar Tests**

**Two-Sample Kolmogorov-Smirnov Test**

**NPar Tests**

**Two-Sample Kolmogorov-Smirnov Test**

**NPar Tests**

**Two-Sample Kolmogorov-Smirnov Test**

**NPar Tests**

**Two-Sample Kolmogorov-Smirnov Test**

**NPar Tests**

**Two-Sample Kolmogorov-Smirnov Test**

**NPar Tests**

**Two-Sample Kolmogorov-Smirnov Test**

**NPar Tests**

**Two-Sample Kolmogorov-Smirnov Test**

**NPar Tests**

**Two-Sample Kolmogorov-Smirnov Test**

**NPar Tests**

**Two-Sample Kolmogorov-Smirnov Test**
